# Kidney Involvement in Primary Sjögren Syndrome: Focus on Tubulointerstitial Nephritis

**DOI:** 10.3390/medicina62102028

**Published:** 2026-10-20

**Authors:** Domenico Giannese, Diego Moriconi, Gaetano La Rocca, Linda Ceccarelli, Paola Migliorini, Ilaria Della Buona, Andres Giannini, Angelo Giovanni Bonadio, Francesco Ferro, Sofia D’Elios, Guido Ricchiuti, Claudia D’Alessandro, Vincenzo Panichi, Chiara Baldini

**Affiliations:** 1 Nephrology Unit, Department of Clinical and Experimental Medicine, Azienda Ospedaliero Universitaria Pisana, 56126 Pisa, Italy; 2 Department of Clinical and Experimental Medicine, University of Pisa, 56126 Pisa, Italy; 3 Rheumatology Unit, Department of Clinical and Experimental Medicine, University of Pisa, 56126 Pisa, Italy; 4 IBD Unit, Department of General Surgery and Gastroenterology, University of Pisa, 56126 Pisa, Italy; 5 Clinical Immunology and Allergy Unit, Department of Clinical and Experimental Medicine, University of Pisa, 56126 Pisa, Italy; 6 Pathology Unit, ASLAL, 15121 Alessandria, Italy; 7 Pediatrics Unit, Department of Clinical and Experimental Medicine, Azienda Ospedaliero Univesitaria Pisana AOUP, 56126 Pisa, Italy; 8 Department of Nephrology and Dialysis, Versilia Hospital, 55041 Lido di Camaiore, Italy

**Keywords:** primary Sjögren syndrome, tubulointerstitial nephritis, kidney biopsy

## Abstract

*Background and Objectives*: Primary Sjögren’s syndrome (pSS) is a systemic autoimmune disease with heterogeneous extraglandular involvement, including the kidney. Tubulointerstitial nephritis (TIN) is the hallmark kidney lesion of pSS, but its clinical and histological features, compared with other causes of TIN, remain incompletely characterized. This study aimed to describe the clinical, laboratory, and histological features of biopsy-proven pSS-associated TIN (pSS-TIN) and to compare them with TIN of other etiologies. *Materials and Methods*: We retrospectively analyzed native-kidney biopsies (2015–2025) with histological diagnosis of TIN, from adult and pediatric patients, excluding cases with neoplasms or concomitant acute glomerular injury. Clinical, laboratory, and histological data of pSS-TIN patients were compared with other TIN etiologies using the Mann–Whitney U test, Fisher’s exact test, and Spearman’s correlation; *p* < 0.05 was significant. *Results*: Among 690 native-kidney biopsies, 35 showed TIN; after excluding cases with glomerular involvement, 14 patients with pSS-TIN and 16 patients with no-pSS-TIN patients were analyzed. All pSS-TIN patients were female, and biopsy occurred after a significantly longer interval from disease diagnosis than patients with no-pSS-TIN (*p* = 0.018). Acute kidney injury was less frequent (*p* = 0.021) and kidney function was better preserved in pSS-TIN, while the urea-to-creatinine ratio was similar between groups. Standard urinalysis was abnormal in only about half of patients in both groups. Longer disease duration correlated with lower eGFR in pSS-TIN (*p* = 0.033). Predominantly chronic histological damage was associated with higher proteinuria (*p* = 0.037) across the entire cohort, and germinal-center-like lymphoid structures were observed in two pSS-TIN patients with extensive acute damage. In our cohort, no statistically significant association was detected between kidney function indices and the severity of chronic damage. *Conclusions*: In this biopsy-based cohort, pSS-TIN showed a predominantly chronic presentation, less acute kidney injury, and a longer diagnostic interval than other TIN forms. Routine urinary and functional parameters may not reliably reflect chronic tubulointerstitial damage, highlighting the importance of careful renal screening and timely nephrological assessment, with kidney biopsy when clinically indicated.

## 1. Introduction

Primary Sjögren’s syndrome (pSS) is a chronic systemic autoimmune disorder that predominantly affects women. It is characterized by lymphocytic infiltration and progressive dysfunction of exocrine glands, as well as a broad spectrum of extraglandular manifestations. Although xerostomia and keratoconjunctivitis sicca remain the hallmark clinical features, it has become increasingly evident that pSS is characterized by heterogeneous systemic involvement. Nearly 40–50% of patients develop extraglandular manifestations involving the lungs, musculoskeletal system, nervous system, skin, and kidney, which substantially contribute to morbidity, long-term organ damage, impaired quality of life, and increased mortality. This evolving understanding has shifted the perception of pSS from a predominantly exocrine disorder to a complex immune-mediated systemic disease requiring multidisciplinary evaluation and management [1,2].

Despite substantial advances in the understanding of systemic immune dysregulation, the mechanisms responsible for organ-specific injury remain incompletely elucidated. Increasing evidence suggests that epithelial cells actively participate in disease initiation and perpetuation rather than representing passive targets of autoimmunity. This concept of “autoimmune epithelitis” has profoundly influenced current pathogenetic models, highlighting reciprocal interactions between epithelial cells, resident immune populations, cytokine networks, and the interferon signature. Importantly, similar pathogenic mechanisms appear to operate in several extraglandular organs, including the kidney, where chronic lymphoplasmacytic infiltration may reproduce many of the histopathological and molecular features observed in salivary glands.

### Kidney Involvement

Among systemic manifestations of pSS, kidney involvement deserves particular attention because of its potential impact on long-term kidney function and overall prognosis. Although clinically overt kidney disease has traditionally been considered an uncommon complication, accumulating evidence suggests that its true prevalence has probably been underestimated. Reported frequencies vary considerably, ranging from approximately 5% to over 10% in large observational cohorts, while prospective studies employing systematic screening strategies have identified subclinical kidney abnormalities in substantially higher proportions of patients [3].

The recognition of kidney disease in pSS has become increasingly important, although it remains challenging. Glomerular involvement, which may be associated with B-cell hyperactivity, has been reported in association with several histopathological entities, such as cryoglobulinemia, membranous nephropathy, IgA nephropathy, focal segmental glomerulosclerosis (FSGS), minimal change disease, C3 glomerulopathy, fibrillary glomerulonephritis, pauci-immune crescentic glomerulonephritis associated with anti-neutrophil cytoplasmic antibodies (ANCA), and AA amyloidosis [4,5].

Tubulointerstitial nephritis (TIN) is the hallmark kidney manifestation of pSS, accounting for approximately 70–80% of biopsy-proven kidney lesions and representing the principal mechanism underlying chronic kidney dysfunction in affected patients. Although clinically overt kidney disease occurs in fewer than 10% of patients, systematic screening for tubular abnormalities has demonstrated a substantially higher prevalence of subclinical kidney involvement, suggesting that TIN is frequently underrecognized because of its insidious clinical course. The discrepancy between clinically apparent and subclinical disease underscores the importance of routine assessment of kidney function and tubular integrity in patients with systemic pSS [4,6,7,8,9]. Kidney biopsy remains the diagnostic gold standard in patients with suspected TIN. It is characterized by diffuse or patchy lymphoplasmacytic infiltration of the interstitium predominantly composed of CD4^+^ T lymphocytes, although activated B cells and plasma cells become increasingly prominent in advanced disease, supporting the concept of “autoimmune epithelitis” [4,6,7]. Histopathological evaluation not only confirms the diagnosis but also provides important prognostic information by assessing the extent of inflammatory activity, tubular atrophy, and interstitial fibrosis, which correlate more closely with long-term kidney outcome than baseline kidney function alone [4,7,10].

The clinical manifestations of TIN primarily reflect tubular dysfunction: distal tubular acidosis is the most common clinical phenotype and results from impaired proton secretion by α-intercalated cells of the collecting duct. Patients may present with hyperchloremic metabolic acidosis, hypokalemia, nephrolithiasis, nephrocalcinosis, osteomalacia, recurrent muscle weakness, or even hypokalemic paralysis. Less commonly, proximal tubular dysfunction may occur, ranging from isolated low-molecular-weight proteinuria to complete Fanconi syndrome. Urinary concentrating defects and glycosuria represent rare but well-recognized manifestations of advanced tubular injury. Importantly, mild forms of TIN may remain asymptomatic, emphasizing the need for a high index of suspicion in patients with otherwise unexplained electrolyte abnormalities [4,7,8,10].

Current treatment strategies are largely based on observational evidence because randomized controlled trials are lacking. Overall, tubulointerstitial involvement represents the prototypical kidney manifestation of pSS and, despite its generally favorable kidney prognosis when recognized early, delayed diagnosis remains common because tubular dysfunction often precedes overt impairment of glomerular filtration [6,7,8]. In this study, we characterized TIN due to primary Sjögren syndrome (pSS-TIN), including its histological and clinical features.

We aimed to investigate the frequency, clinical presentation, laboratory findings, and histological features of biopsy-proven pSS-TIN and to compare them with TIN associated with other diseases. Further studies will be needed to confirm the results.

## 2. Materials and Methods

We retrospectively analyzed patients with biopsy-proven pSS-TIN evaluated between 2015 and 2025 who had been diagnosed with pSS according to the 2016 ACR/EULAR guidelines before kidney histology. Only native-kidney biopsies with histological confirmation of TIN were included.

Clinical, laboratory, and histological characteristics were compared with those of patients with TIN associated with diseases or clinical conditions, with the aim of identifying features that may distinguish pSS-TIN from other forms of TIN.

The inclusion criteria were:

- Diagnosis of TIN
- Absence of neoplasm
- Availability of sufficient clinical data and absence of major etiological confounding factors
- Absence of acute glomerular injury

All of the biopsies were performed in accordance with good clinical practice for the following indications:

- Proteinuria
- Unexplained acute or chronic kidney damage
- Urinary abnormalities

Pediatric patients were eligible for inclusion.

### Statistical Analysis

Continuous variables are reported as means and standard deviations, whereas categorical variables are presented as counts and percentages. Comparisons between patients with pSS-TIN and those with no-pSS-TIN were performed using the Mann–Whitney U test for continuous variables and the Fisher’s exact test for categorical variables. Associations between ordinal histological scores and continuous clinical or laboratory variables were assessed using Spearman’s rank correlation coefficient. Bivariate analysis was performed for a limited number of patients. No adjustment for multiple comparisons was applied; therefore, all inferential analyses should be considered exploratory. Missing data were not imputed, and variable-specific denominators are reported where applicable.

All tests were two-sided, and *p*-values < 0.05 were considered statistically significant. Analyses were performed using IBM SPSS version 23.

## 3. Results

**Figure 1:**
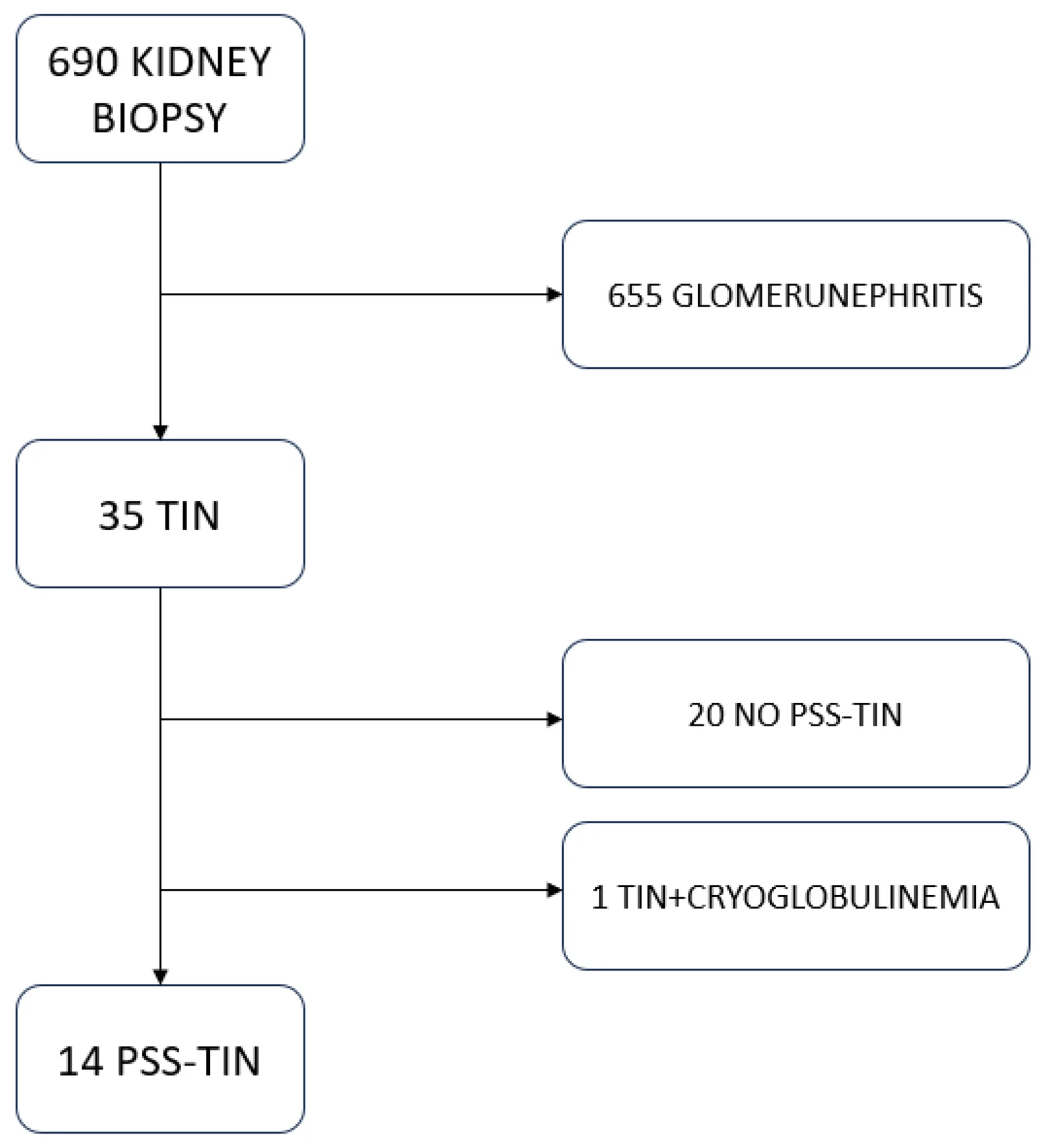
Diagnosis of TIN due to primary Sjögren syndrome (pSS).

Among 690 native-kidney biopsies, 35 showed TIN. Fifteen cases were associated with pSS; one patient with concomitant cryoglobulinemic glomerular involvement was excluded, leaving 14 patients with isolated pSS-TIN for analysis (Figure 1).

We compared pSS-TIN with other forms of TIN with respect to clinical presentation, laboratory findings, and histological features. Four of the 20 no-pSS-TIN cases were excluded because of concomitant glomerular involvement, leaving 16 patients for the comparative analysis. The causes of TIN are summarized in Table 1.

### 3.1. Demography

All patients with pSS-TIN were women and younger than those with no pSS-TIN, although the difference did not reach statistical significance. The data are summarized in Table 2.

The patients with pSS-TIN were all women, in contrast to the male predominance seen in other forms of TIN. Furthermore, regarding the diagnosis of the disease—whether assessed as a serological diagnosis, for the pSS according with ACR/EULAR criterias, as a systemic extrarenal presentation, or as the onset of clinical symptoms—patients with pSS-TIN underwent a kidney biopsy after a significantly longer period of time.

### 3.2. Clinical Features

pSS-TIN patients underwent a kidney biopsy primarily due to proteinuria, whereas among patients with other forms of TIN, cases of acute kidney injury (AKI) were much more common. AKI was defined in accordance with the KDIGO guidelines. All patients with this presentation had a known increase in baseline creatinine levels:

- An increase in serum creatinine of ≥0.3 mg/dL (≥26.5 μmol/L) within 48 h, or
- An increase in serum creatinine to ≥1.5 times the baseline value, known or presumed to have occurred within the previous 7 days.

None of the patients experienced a decrease in urine output.

Consistent with acute damage, patients with no-pSS-TIN had worse kidney function and elevated blood urea nitrogen levels. However, despite the differences in kidney function, the urea-to-creatinine ratio was approximately 30. In both groups, the standard urine analysis was abnormal in only about half of the patients (Table 3).

We did not find any correlation between the level of proteinuria and kidney function, eGFR (*p* = 0.7) and creatinine clearance (*p* = 0.8).

Moreover, we analyzed the urinary data. In particular, we focused on urinary specific gravity, the presence of proteinuria and glycosuria, and urinary sediment. We did not find any difference between pSS-TIN and other forms of TIN. One consistent finding was urinary isosthenuria, a typical sign of tubular dysfunction, although it does not distinguish between acute and chronic damage. An interesting finding was, in general, a pathological urinalysis, defined as the presence of at least one of the following evaluated parameters: presence of proteinuria, glycosuria, or urinary sediment. We found that it was positive in fewer than ¾ of the patients (Table 4).

When analyzing the time elapsed between clinical diagnosis and kidney biopsy, there is a significant correlation between diagnosis, kidney biopsy and a reduction in eGFR. In particular, it is noted that a longer duration of the disease is a significant factor in the reduction of kidney function (*p* = 0.033, r = −0.591). The time interval from onset to histological diagnosis did not correlate with proteinuria.

### 3.3. Histology

Histology was classified as acute or chronic. A score was assigned to objectively assess the extent of damage in both conditions: mild (<25% of the parenchyma), moderate (25–50% of the parenchyma) and severe (>50% of the parenchyma) (Table 5). In case of acute damage, an overall assessment was made of the extent of involvement in the sample with respect to the presence of individual lesions, such as tubulitis, acute interstitial infiltrate, and edema.

The main histological features were consistent with those reported in the literature, in particular the presence of eosinophils in cases of drug-induced damage and the predominance of IgG4-positive plasma cells in patients with IgG4-RD. No other relevant differences in inflammatory infiltrates were observed among patients with TIN. Germinal-center-like lymphoid structures were observed in two patients with pSS-TIN. In particular, two patients exhibited the typical characteristics, with CD20-positive elements at the center surrounded by CD3-positive elements. In both biopsies, there was also a high degree of acute damage (>50% of the tissue).

We found no significant associations between the severity of acute and chronic damage and creatinine, eGFR, creatinine clearance and proteinuria in the entire cohort, in pSS-TIN and in non-pSS-TIN. When patients were stratified according to the predominant type of damage, chronic or acute, proteinuria was higher in the case of chronic damage (*p* = 0.037) but there were no differences in kidney function (creatinine, eGFR or CrCl) for the entire cohort. This evidence was not confirmed in the pSS-TIN cohort. In non-pSS-TIN patients, predominant acute damage was associated with worse kidney function, as assessed by eGFR (*p* < 0.001) and CrCl (*p* = 0.017).

In cases of moderate/severe chronic damage (score 2–3), a reduction in kidney function was identified, as measured by eGFR; however, this finding was not statistically significant (*p* = 0.067). Only in this case, in light of the result being on the borderline of statistical significance, a bivariate analysis was performed, confirming the finding even after adjustment for acute damage (mild vs. moderate/severe) (*p* = 0.076); the same lack of significance was also evident using CrCl (*p* = 0.17). Therefore, routine clinical measures of kidney function do not allow the chronicity of kidney involvement to be predicted in cases where TIN is suspected.

## 4. Discussion

TIN is a complex and challenging diagnosis; it is therefore recognized as an underrecognized kidney condition. In clinical practice the evaluation of kidney damage is based on eGFR and albuminuria [11], which are mainly glomerular markers and do not evaluate tubular damage [12]. However, it is well known that tubulointerstitial fibrosis is highly predictive of progression of kidney disease [13]. Nowadays, new biomarkers are available to detect tubular dysfunction [14] but these are not validated in pSS. Promising studies on specific biomarkers in TIN for pSS have identified the presence of urinary CD4+ T helper cells as a marker associated with the severity of kidney involvement. However, the data relate to a small cohort of patients. Therefore, these findings remain promising and require verification at a large scale [15]. The cohorts reported in the literature often consist of multicenter studies in various populations, and kidney involvement was interpreted as TIN in the absence of a biopsy. Consequently, the findings reported in these papers are subject to interpretative limitations [16,17,18]. In the cases described, immunosuppressive therapy has improved kidney function even in the presence of fibrosis. However, there is no benefit from treatment with steroids or other immunosuppressive agents. Our single-center study investigated the overall histological presentation of TIN in pSS, using a control group to gain a better understanding of the data. When compared with the group of patients with TIN due to other causes, pSS-TIN showed a distinct clinical pattern: it mainly affected women, consistent with the literature, and was predominantly characterized by proteinuria and abnormal urine test results. Indeed, acute kidney injury—a presentation that often leads patients with tubulointerstitial damage to undergo a kidney biopsy—is statistically less common in PSS-TIN than in other forms of TIN undergoing histological evaluation, potentially contributing to delayed recognition of kidney involvement in pSS. This aspect further highlights the diagnostic challenge. It is important to note that the design of our study has a limitation related to the heterogeneity of the non-pSS-TIN group, which includes patients with different etiologies. Furthermore, given the nature of this study, we highlight limitations related to demographic differences in the study population, primarily due to its small sample size. It should be noted that this is an exploratory approach and that further evidence is needed to confirm these findings. The urea-to-creatinine ratio was approximately 30, similar in both groups. This value, as a proposed but unvalidated potential marker of tubulointerstitial damage [19], does not distinguish between the two forms. While AKI warrants close monitoring and a reasonable acceptance of the risks associated with a kidney biopsy, more subtle abnormalities are more difficult to detect. Indeed, in screening carried out using a urine test alone, no pathological findings were observed in 30% of the patients in our cohort. Findings of proteinuria measured in 24-h urine samples and electrolyte abnormalities not attributable to drug therapy or other causes are highly significant; these are often identified in our patients as a sign of tubular dysfunction in regulation. However, in our experience, the presence and the amount of proteinuria or an abnormal urine analysis are not associated with the extent of the damage, whether acute or chronic. Therefore, these findings are useful for identifying patients who might benefit from further evaluation, including a kidney biopsy. In this cohort, these laboratory parameters did not reliably reflect the extent of histological kidney involvement. Our histological analysis provides an additional clinically relevant observation. Patients with moderate-to-severe chronic damage exhibited lower eGFR values, although the association did not reach statistical significance, including after adjustment for acute damage (*p* = 0.076). This may reflect the limited sample size and the heterogeneity of TIN, but it also illustrates the limitations of serum creatinine and eGFR as surrogates of structural tubulointerstitial damage. The association between longer disease duration and lower eGFR in pSS-TIN may suggest a relationship between cumulative kidney injury and functional impairment. Based on our experience, we found that a correct indication for kidney biopsy requires combining quantitative and qualitative determinations of proteinuria associated with blood findings (kidney function and electrolyte abnormalities), rather than relying on a single value. We suggest considering not only urinalysis, as recommended, for example, in guideline of pSS, but all the urinary and blood parameters derived from our experience to perform accurate screening, according to the discrepancies between laboratory findings and the extent of histological kidney injury (acute or chronic, segmental or diffuse) described in our cohort. The potential value of this approach is that it may help identify patients for whom improved nephrological follow-up and consideration of kidney biopsy are indicated, and therefore detect possible acute TIN, in order to intensify and/or personalize immunosuppressive therapy, in light of the new therapeutic options available, as suggested in the literature [16]. This aspect, as suggested by our findings, is of particular importance given that when TIN is adequately diagnosed, assessed histologically and, above all, treated with the appropriate immunosuppressive regimen, kidney function and proteinuria may improve, which, in our opinion, protects against end-stage kidney disease. The importance of a kidney biopsy lies, in fact, primarily in distinguishing acute TIN—which requires specific treatment—from chronic interstitial fibrosis, which is characterized by irreversible damage and for which only monitoring and supportive care are indicated. The significance of kidney histology, to be performed according to appropriate clinical evaluation, is further underscored by the low predictive ability of urinalysis alone, which is insufficient, as shown in our paper. When clinically indicated, a kidney biopsy, when it can be performed, can accurately characterize the extent of TIN, representing the cornerstone for assessing kidney involvement and selecting the most appropriate treatment. It is important to note that our approach, which quantifies acute and chronic damage based on findings in the literature from other clinical contexts not specific to pSS-TIN [20,21], has not been validated and will require further studies to assess its clinical utility in this specific disease context. If the screening of TIN is performed only with urinalysis, as often occurs, the risk of underdiagnosis or underestimation of the tubulointerstitial involvement may be real. Our findings suggest that diagnosing TIN in patients with autoimmune diseases remains a challenge and the indication for kidney biopsy should consider a combination of quantitative and qualitative urinary findings together with kidney function and electrolyte abnormalities, rather than relying on a single parameter, to avoid diagnostic delays and chronic kidney damage.

## 5. Conclusions

In this biopsy-based cohort, pSS-TIN showed a predominantly chronic presentation, with less AKI and a longer diagnostic interval than other forms of TIN. Routine urinary, functional parameters and laboratory findings may not reliably reflect chronic tubulointerstitial damage, supporting careful kidney screening and timely nephrological assessment and, if necessary, kidney biopsy in patients with pSS. Given that this was a single-center study with a limited sample size, further studies will be needed to confirm the results.

## Figures and Tables

**Table 1:** The causes of interstitial nephritis. IBD: inflammatory bowel disease, IgG4-RD: IgG4-related disease, AAV: ANCA-associated vasculitis, TINU: tubulointerstitial nephritis and uveitis.

Total	16
Drug	5 (31.25%)
IBD	4 (25%)
IgG4-RD	3 (18.75%)
AAV	3 (18.75%)
TINU	1 (6.25%)

**Table 2:** Demography of the cohort.

	Total N = 30	pSS-TIN N = 14	no-pSS-TIN N = 16	*p*
Female (Male)	17 (13)	14 (0)	3 (13)	**0.0001**
Caucasian (other)	28 (2)	12 (2)	16 (0)	0.2
Age at the biopsy	55 ± 14.9	51 ± 15	58.5 ± 15	0.08
From diagnosis to biopsy (months)	30 ± 50	50 ± 68	12 ± 10	**0.018**

Values in bold indicate statistically significant results (*p* < 0.05).

**Table 3:** Clinical and laboratory findings. AKI: acute kidney injury, eGFR: estimated glomerular filtration rate, CrCl: creatinine clearance.

	Total	pSS-TIN	No-pSS-TIN	*p*
AKI	11 (36.6%)	2 (14.2%)	9 (56.2%)	**0.021**
Creatinine (mg/dL)	2.3 ± 1.4	1.4 ± 0.56	3 ± 1.5	**<0.001**
Urea (mg/dL)	68.6 ± 40.7	43 ± 17.7	91 ± 42.3	**<0.001**
Urea/Creatinine	30.9 ± 6.4	31 ± 6.5	30.8 ± 6.6	0.91
eGFR (EPI) (mL/min/1.73 m^2^)	42.1 ± 28	58.1 ± 29.3	28.1 ± 18	**0.001**
CrCL (ml/min)	54.2 ± 31	67.6 ± 29.5	41.9 ± 28.1	**0.04**
Proteinuria (mg/24 h)	815 ± 652	711 ± 533	906 ± 747	0.21
Pathological urinalysis	21 (70%)	11 (78.6%)	10 (62.5%)	0.4
Electrolytes abnormalities	29 (96.6%)	14 (100%)	15 (93.75%)	1

Values in bold indicate statistically significant results (*p* < 0.05).

**Table 4:** The urinary data. The table lists the numbers of patients with a positive finding in the entire cohort, among patients with pSS-TIN and among the others. USG: urinary specific gravity.

	Total	pSS-TIN	no-pSS-TIN	*p*
USG	1009 ± 3	1009 ± 3	1009 ± 2	0.67
Proteinuria	17 (56%)	7 (50%)	10 (62.5%)	0.7
Glycosuria	4 (13.3%)	2 (14.3%)	2 (12.5%)	1
Urinary sediment	14 (46.6%)	5 (35.7%)	9 (56.5%)	0.7
Urinalysis	21 (70%)	11 (78.6%)	10 (62.5%)	0.4

**Table 5:** The severity of the damage: 0: absence, 1: mild (<25%), 2: moderate (25–50%), 3: severe (>50%). pSS-TIN: tubulointerstitial nephritis due to Primary Sjögren’s syndrome, AAV: ANCA-associated vasculitis, IgG4-rd: IgG4-related disease, IBD: inflammatory bowel disease, TINU: tubulo interstitial nephritis and uveitis, Drug: drug-induced TIN.

Disease	Acute Damage	Chronic Damage
pSS-TIN	3	1
pSS-TIN	2	3
pSS-TIN	1	1
pSS-TIN	0	2
pSS-TIN	1	1
pSS-TIN	3	3
pSS-TIN	3	1
pSS-TIN	2	2
pSS-TIN	1	0
pSS-TIN	3	2
pSS-TIN	3	3
pSS-TIN	2	1
pSS-TIN	3	2
pSS-TIN	1	1
AAV	1	0
AAV	1	2
AAV	1	2
IgG4-rd	2	2
IgG4-rd	3	2
IgG4-rd	3	1
IBD	2	0
IBD	0	2
IBD	1	2
IBD	2	2
TINU	3	1
DRUG	3	1
DRUG	1	2
DRUG	1	3
DRUG	1	2
DRUG	1	1

## Data Availability

The original contributions presented in this study are included in the article. Further inquiries can be directed to the corresponding author.
